# Initiating change locally in bullying and aggression through the school environment (INCLUSIVE) trial: update to cluster randomised controlled trial protocol

**DOI:** 10.1186/s13063-017-1984-6

**Published:** 2017-05-25

**Authors:** Chris Bonell, Anne Mathiot, Elizabeth Allen, Leonardo Bevilacqua, Deborah Christie, Diana Elbourne, Adam Fletcher, Richard Grieve, Rosa Legood, Stephen Scott, Emily Warren, Meg Wiggins, Russell M. Viner

**Affiliations:** 1 London School of Hygiene & Tropical Medicine, Keppel Street, London, WC1E 7HT UK; 2 UCL Great Ormond Street Institute of Child Health, 30 Guilford St, London, WC1N 1EH UK; 3 UCL Hospitals NHS Foundation Trust, 250 Euston Rd, London, NW1 2PG UK; 4 Cardiff School of Social Sciences, Cardiff University, Glamorgan Building, King Edward VII Avenue, Cardiff, CF10 3WT UK; 5 Institute of Psychiatry, Kings College London, 16 De Crespigny Park, London, SE5 8AF UK; 6 UCL Institute of Education, 20 Bedford Way, London, WC1H 0AL UK

**Keywords:** Bullying, Cluster randomised trial, School intervention, Violence prevention, Adolescent

## Abstract

**Background:**

Systematic reviews suggest that multi-component interventions are effective in reducing bullying victimisation and perpetration. We are undertaking a phase III randomised trial of the INCLUSIVE multi-component intervention. This trial aims to assess the effectiveness and cost-effectiveness of the INCLUSIVE intervention in reducing aggression and bullying victimisation in English secondary schools. This paper updates the original trial protocol published in 2014 (Trials 15:381, 2014) and presents the changes in the process evaluation protocol and the secondary outcome data collection.

**Methods:**

The methods are summarised as follows.

Design: cluster randomised trial.

Participants: 40 state secondary schools. Outcomes assessed among the cohort of students at the end of year 7 (*n* = 6667) at baseline.

Intervention: INCLUSIVE is a multi-component school intervention including a social and emotional learning curriculum, changes to school environment (an action group comprising staff and students reviews local data on needs to review rules and policies and determine other local actions) and staff training in restorative practice. The intervention will be delivered by schools supported in the first two years by educational facilitators independent of the research team, with a third intervention year involving no external facilitation but all other elements.

Comparator: normal practice.

Outcomes:

Primary: Two primary outcomes at student level assessed at baseline and at 36 months:

1. Aggressive behaviours in school: Edinburgh Study of Youth Transitions and Crime school misbehaviour subscale (ESYTC)
2. Bullying and victimisation: Gatehouse Bullying Scale (GBS)

Secondary outcomes assessed at baseline, 24 and 36 months will include measures relating to the economic evaluation, psychosocial outcomes in students and staff and school-level truancy and exclusion rates.

Sample size: 20 schools per arm will provide 90% power to identify an effect size of 0.25 SD with a 5% significance level.

Randomisation: eligible consenting schools were randomised stratified for single-sex versus mixed-sex schools, school-level deprivation and measures of school attainment.

**Discussion:**

The trial involves independent research and intervention teams and is supervised by a Trial Steering Committee and a Data Monitoring Committee.

**Trial registration:**

Current Controlled Trials, ISRCTN10751359. Registered on 11 March 2014.

## Changes to the original protocol

### Amendment 1

The team suggested changes to the process evaluation section of the original protocol [1]. These changes were endorsed by our Trial Steering Committee (TSC) and approved by UCL Research Ethics Committee (5/10/2015, ref 5248/001).

The deviations from the original protocol and rationales for these changes are provided in Table 1.

**Table 1:** Changes to the original protocol approved in Amendment 1

	Change to the original protocol	Rationale behind the change
Staff telephone interviews	The protocol originally included conducting interviews with 1 member of the school senior leadership team (SLT) and 2 teaching staff annually (years 1–3) across 40 schools (intervention and control). These were completed as per the protocol for year 1. We do not intend to conduct staff telephone interviews in year 2. We will conduct interviews with 1 SLT member in each of the 40 schools (intervention and control) in year 3. Control schools will be interviewed in term 1, and intervention schools will be interviewed in term 3	Interviews in year 2 were considered unnecessary since we are already collecting other data (e.g. via interviews with action team members, curriculum surveys, focus groups) on how the intervention is progressing in intervention schools. Interviews in years 3 and 1 are sufficient to assess provision in control schools. Some control schools have also reported overburden following year 1 interviews, so we have reduced the number of interviews for year 3. Resources are being re-directed to in-depth case studies of intervention schools (and away from superficial data collection across all schools)
Researcher observations of curriculum delivery	We originally intended to observe *n* = 1 curriculum session in each school but are now using a curriculum survey circulated to the intervention curriculum co-ordinator in each school to assess what was delivered, how and when. Interviews with curriculum leads will also be conducted	The lead intervention facilitator advised us that observations would create an excessive administrative burden for schools, and our modified approach provides fuller data on implementation of this component
Action group meeting observations	This will be done in *n* = 10 schools per year rather than *n* = 20 schools	We are collecting substantial amounts of other data on action groups via facilitator diaries and collection of all action group documentation. The observations act as a check on the validity of diary data provided by facilitators and do not need to be done across all 20 schools each year. We will re-direct the researcher time that would have been spent on this to more in-depth data from case study schools
Case study schools	The protocol originally specified case studies in *n* = 4 control schools and *n* = 4 intervention schools. We now plan to conduct case studies in *n* = 6 intervention schools only	Control schools have complained about being overburdened with fieldwork requests, and we think that asking too much of them may threaten follow-up rates in the trial. The main purpose of the case studies is to capture data on intervention mechanisms. Case studies of control schools will not be informative about mechanisms, but will only inform us about what activities constitute the control condition in the trial, which we are already collecting across all control schools. We have re-directed resources so that we are doing more work in intervention schools (*n* = 6 schools as case study sites; conducting 1 focus group with staff, 2 focus groups with students and 2 interviews with students who were involved in restorative practices in each school)

The main reason for changing the protocol is to limit the data collection’s burden imposed on schools and re-direct the resources to in-depth data analysis and additional data collection collected from intervention schools.

### Amendment 2

The study executive team thought it would be in the interest of the study to add a question on bullying perpetration. The change was supported by our TSC and has been approved by UCL Research Ethics Committee (23/03/2016, ref 5248/001). This added a new secondary outcome to the study and an additional question in the students’ questionnaire delivered in the year 2 and year 3 follow-up surveys. The protocol has been amended accordingly in the secondary outcome section, and with a minor correction in the statistical section.

The question is taken from the Centers for Disease Control and Prevention (CDC) guidance document on bullying measures [2]. The only measure that it recommends that focuses on specific occasions of recent bullying perpetration is the Modified Aggression Scale Bullying subscale (Cronbach’s alpha = 0.83) [3]. This is an existing, established measure with evidence of reliability.

### Amendment 4

The current approved protocol (v1.5) had some details missing in the Process Evaluation section of the protocol. These details were in our Process Evaluation (PE) protocol, approved by our TSC, so the team thought it important to align the main protocol with the PE protocol by adding more details in the main section, new version 1.6. The amendment was approved on 10/10/2016.

The additional details added:

- Section *Trial arm fidelity*: “termly (from year 3 annual) restorative practice surveys (*n* = 20)” and “We will also draw on administrative documents (e.g. minutes, attendance sheets, training satisfaction feedback)”
- Section *Reception and responsiveness*: “We will also interview *n* = 2 students involved in restorative practice sessions per year in each case study school.”

## Abbreviations

CDC: Centers for Disease Control and Prevention
DMC: Data Monitoring Committee
ESTYC: Edinburgh Study of Youth Transitions and Crime
GBS: Gatehouse Bullying Scale
TSC: Trial Steering Committee
